# Total Antioxidant Capacity: Biochemical Aspects and Clinical Significance

**DOI:** 10.3390/ijms241310978

**Published:** 2023-07-01

**Authors:** Andrea Silvestrini, Elisabetta Meucci, Bianca Maria Ricerca, Antonio Mancini

**Affiliations:** 1 Dipartimento di Scienze Biotecnologiche di Base, Cliniche Intensivologiche e Perioperatorie, Università Cattolica del Sacro Cuore, 00168 Rome, Italy; 2 Fondazione Policlinico Universitario Agostino Gemelli (IRCCS), 00168 Rome, Italy; 3 Dipartimento di Diagnostica per Immagini, Radioterapia Oncologica ed Ematologia, Università Cattolica del Sacro Cuore, 00168 Rome, Italy; 4 Dipartimento di Medicina e Chirurgia Traslazionale, Università Cattolica del Sacro Cuore, 00168 Rome, Italy

**Keywords:** oxidative stress, total antioxidant capacity, obesity, metabolic syndrome, redox balance

## Abstract

Despite the physiological role of oxidant molecules, oxidative stress (OS) could underlie several human diseases. When the levels of antioxidants are too low or too high, OS occurs, leading to damage at the molecular, tissue and cellular levels. Therefore, antioxidant compounds could represent a way to modulate OS and/or to maintain proper redox balance. This review provides an overview of the methods available to assess total antioxidant capacity (TAC) in biological systems to elucidate the correct terminology and the pathophysiological roles. The clinical context is fundamental to obtain a correct interpretation of TAC. Hence, we discuss metabolic syndrome and infertility, two clinical conditions that involve OS, including the potential prognostic role of TAC evaluation in monitoring antioxidant supplementation. This approach would provide more personalised and precise therapy.

## 1. Redox Homeostasis, Oxidative Stress (OS) and Total Antioxidant Capacity (TAC)

**Figure 1:**
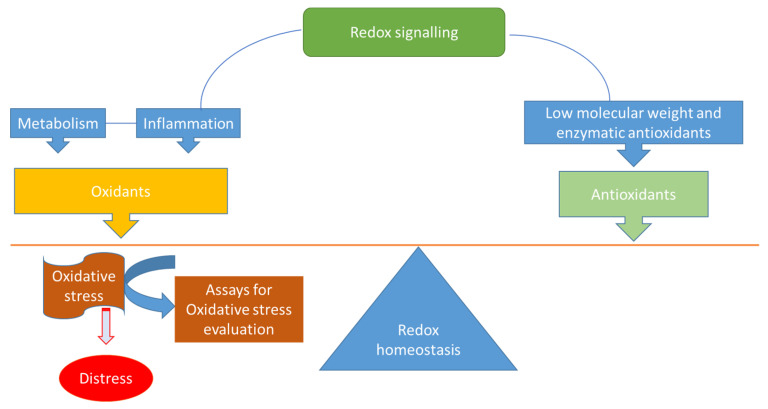
Redox homeostasis: the role of oxidants/antioxidants in the disruption/maintenance of redox homeostasis. If the levels of oxidants produced during metabolism and/or inflammation are too high, then oxidative stress occurs and can be evaluated by using several assays.

There is compelling evidence highlighting that redox homeostasis is crucial for cells and indeed the entire body: disruption can alter practically all biological processes (e.g., metabolism, cell proliferation, differentiation, cellular senescence and autophagy) [1]. Several biological processes in humans (e.g., breathing, digestion, hormone biosynthesis and xenobiotic metabolism) can produce oxidants through redox signalling. Importantly, reactive oxygen species (ROS) is a generic term for a large family of oxidants derived from molecular oxygen [1,2]. In this review, we avoid using ROS, preferring the more adequate term “oxidant”. This physiological production of oxidants is regularly inactivated by antioxidant defences, thus balancing redox homeostasis and protecting against damage (Figure 1). If these antioxidant defences are insufficient or redox signalling is altered, then oxidants are able to damage the organism—for example, oxidants can destroy the cell membrane and block the action of major enzymes and processes, a phenomenon also referred to as distress (Figure 1) [1,2]. While this situation appears to be paradoxical, it is important to understand that the oxidants implicated as physiological messengers in redox signalling (e.g., hydrogen peroxide (H_2_O_2_)) and the oxidants responsible for the detrimental oxidation of biomolecules (e.g., the hydroxyl radical (^•^OH)) are not the same [2].

An important question to ask is what exactly are oxidants? They are reactive compounds containing oxygen that can play several beneficial or detrimental roles in an organism. The main oxidants are the superoxide radical (^•^O_2_^−^), ^•^OH and H_2_O_2_, all of which result from the reduction of molecular oxygen [3]. Thus, these molecules are constantly generated as metabolic by-products in biological systems.

It should also be noted that low concentrations of oxidants are required for a plethora of cellular processes, including cell signalling, homeostasis, apoptosis and defence against pathogens, among others. This type of cellular stress, which maintains homeostasis by protecting against damage, is referred to as eustress. In fact, ambient intracellular oxidants (i.e., H_2_O_2_) can regulate various signal transduction pathways by reversibly oxidising cysteine residues in several proteins, thus modulating their activities [3]. These proteins, denoted as “redox switches”, include kinases, protein phosphatases and transcription factors that help regulate various metabolic pathways [1,2,3]. Furthermore, under physiological conditions, defence against the harmful effects of oxidants is achieved by maintaining a balance between oxidants and antioxidants. Thus, defence mechanisms that maintain redox homeostasis can outweigh oxidant-induced damage [4]. Exceeding antioxidants intake may develop an increased “antioxidative stress”, since it diminishes the radicals with a beneficial physiological role [4]. Normally, the processes that produce oxidants are counteracted by antioxidant defences—but what happens if these systems do not work properly? Oxidants can be present in excess concentrations, and free radicals and oxidants give rise to a phenomenon known as OS. This harmful process can negatively affect cellular structures, especially membranes, lipids, proteins and nucleic acids [5]. OS leads to irreversible macromolecular changes in addition to the disruption of normal cellular signalling. According to their activity, antioxidants can be categorised into low-molecular weight and enzymatic antioxidants. The first class of antioxidants (e.g., uric acid, vitamin E and glutathione) inhibit the chain reaction of oxidation, acting as acceptors of free radicals and thus preventing/decreasing radical propagation. This class of antioxidants is also referred to as the “chain breakers” (Table 1).

**Figure 2:**
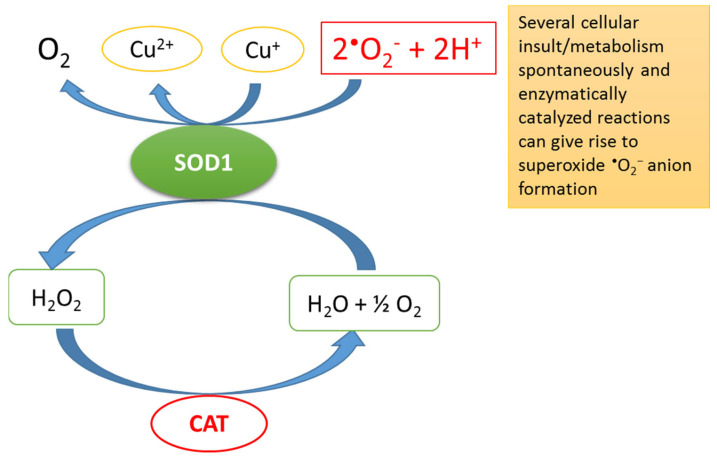
The reaction catalysed by superoxide dismutases (SODs). These enzymes dismutate the superoxide anion radical (^•^O_2_^−^ + 2H^+^), producing hydrogen peroxide (H_2_O_2_) from water and molecular oxygen with the help of copper. Subsequently, catalase (CAT) (or other enzymes) can convert H_2_O_2_ into water and oxygen. SOD1 = super oxide dismutase 1 (mitochondrial intermembrane isoforme); CAT = catalase; Cu = copper.

The enzymatic antioxidants mainly include superoxide dismutases (SODs), glutathione peroxidase, thioredoxin reductase and catalase (CAT). These enzymes interact with their substrates and convert hydroperoxides into inactive non-radical species. SODs are ubiquitous in all organisms and are one of the body’s first enzymatic antioxidant defences [6]. In fact, SODs are at the frontline of the defence against oxidants because they dismutate ^•^O_2_^−^ into molecular oxygen and H_2_O_2_, diminishing the concentration of ^•^O_2_^−^—the most dangerous free radical in all cell compartments—and thus preventing OS (Figure 2) [6].

Accordingly, antioxidant activity is the ability of a compound or an enzyme to diminish the production of oxidants or reactive species. Biomarkers of OS and their possible quantitative evaluation are important tools in the assessment of both disease status and the health-enhancing effects of antioxidants in humans [6]. Over the past decade, a number of quantitative methods have been developed to measure excess oxidants and redox signalling [7]. Several OS biomarkers have been proposed as the gold standard. Moreover, there is no generally accepted method to quantify antioxidant molecules in the presence of other interfering compounds—and this potential drawback limits the effectiveness of antioxidant assays [8]. Unfortunately, none of the OS biomarkers reflect the oxidation products of all biological molecules (e.g., lipids, proteins and nucleic acids). Some of the commonly used antioxidant biomarkers, including TAC (i.e., non-enzymatic markers: vitamins, glutathione and uric acid) and enzymatic antioxidants (e.g., CAT, glutathione peroxidase and SODs), have been used to develop new assays that provide quantitative measures of redox signalling [7]. Nevertheless, measuring TAC remains one of the most widely used methods to quantify the possible oxidant-buffering capacity of a sample. Moreover, TAC assays are frequently used to rank antioxidants and to identify structure–activity relationships. Therefore, TAC assays are often employed to search for unknown antioxidants in complex mixtures.

## 2. Assays

In general, TAC assays employ a thermal radical generator to generate a steady flux of radicals in solution. The presence or addition of an antioxidant competes with probes (i.e., the substrates) for the radicals, and inhibits or retards the oxidation of the probe. TAC assays have been extensively used on biological samples to estimate the extra-cellular non-enzymatic antioxidants. The plasma concentrations of different antioxidants can be measured in laboratories by using these assays. The tests that measure antioxidants are categorised as direct or indirect. Assays that measure TAC directly are based on the ability to inhibit the oxidation of a substance. The most frequently used direct assay is the Trolox equivalent antioxidant capacity (TEAC) test, with some modifications mainly based on the time interval used for the measurement (e.g., frequently for 1 min) and the radical formation [9]. Trolox (6-hydroxy-2,5,7,8-tetramethylchroman-2-carboxylic acid) is an analogue of vitamin E. Other methods that report the value directly include the oxygen radical absorbance capacity (ORAC) and total radical trapping antioxidant parameter (TRAP) assays. The most widely used indirect methods are the ferric-reducing ability of plasma (FRAP) and the cupric-reducing antioxidant capacity (CUPRAC) assays. Both are based on determining the ability of a sample to reduce a metal complex (i.e., Fe^3+^ and Cu^2+^, respectively). An important difference between these assays is that while the TEAC, FRAP and CUPRAC assays are carried out using a spectrophotometer, the ORAC and TRAP assays require a fluorimeter. Several methods for detecting “intracellular oxidants” in cell cultures use the 2,7′-dichlorodihydrofluorescein *diacetate* (DCFH-DA) as a probe in a fluorescence assay. Other methods employ nitroblue tetrazolium (NBT) chloride. NBT, a water-soluble nitro-substituted aromatic tetrazolium molecule, is an artificial electron acceptor that enters within the targeted cells following their incubation in NBT solution. Intracellularly, NBT (or DCFH-DA probe) interacts with superoxide, which produces water-insoluble and stable formazan crystals (formazan precipitates after reacting) that can be detected by microscopy for the in situ localization of oxidants, for instance in the sperm pellet. Importantly, all of these assays involve the following components: (1) a molecular probe that absorbs ultraviolet (UV) light or fluoresces to monitor the reaction development, (2) an azo-radical initiator and (3) an antioxidant (already present in the sample or added exogenously) (Table 2).

### 2.1. The TEAC Assay

The TEAC assay was reported first by Miller [10]. The TEAC assay, frequently reported as TAC, is based on the inhibition of the absorbance by antioxidants of the radical cation of 2,2′-azinobis(3-ethylbenzothiazoline 6-sulfonate) (ABTS^•+^), which has a characteristic absorption spectrum with maxima at 415, 660, 734 and 820 nm. The original version of ABTS^•+^ was developed and sold by Randox Laboratories (Crumlin, UK). The reaction of metmyoglobin with H_2_O_2_ generates the radical species that is formed by the interaction between ABTS^+^ and ferrylmyoglobin. Thus, the TEAC assay measures the ability of a compound to reduce ABTS^•+^, although the compound under analysis can also reduce ferrylmyoglobin radicals. The stability of ABTS^•+^ certainly does not make it a pro-oxidant. The intra- and inter-assay coefficients of variation (CV) of the original TEAC assay were reported to be 0.54–1.59% and 3.6–6.1%, respectively [11]. A modification of the TEAC assay uses ABTS^•+^ pre-formed by the oxidation of ABTS with potassium persulfate (K_2_S_2_0_8_). Pre-formed ABTS^•+^ is more stable when stored in the dark at room temperature; hence, this assay has been improved [12]. ABTS is oxidised by metmyoglobin and H_2_O_2_ into ABTS^•+^, a characteristic blue-green chromophore with a maximal absorption at 734 nm [13]. When antioxidants are added or present in the samples, ABTS^•+^ is reduced to ABTS and loses it colour. Therefore, this method also spectrophotometrically follows the discolouration of the stable radical to measure the relative antioxidant ability of the samples [10]. This assay is often referred to as the TEAC method, because the reaction rate is usually calibrated with Trolox [14] as an antioxidant standard. Moreover, the ABTS assay has an advantage over other assays because ABTS is freely soluble in both organic and aqueous solvents, so it is applicable to both hydrophilic and lipophilic antioxidants [15]. Overall, the major advantages of ABTS/TEAC are that it is simple to operate, reproducible, and can be used in multiple media to determine both the hydrophilic and lipophilic antioxidant capacity of biological fluids.

### 2.2. The ORAC Assay

The ORAC assay was initially developed by Cao [16]. It assesses the effect of presumed antioxidants by measuring fluorescence quenching. The ORAC assay directly measures the inhibition of a radical reaction, as well as the degree of inhibition, through the addition of a molecular probe. In fact, this assay measures the antioxidant’s ability to inhibit peroxyl radical-induced oxidation and, consequently, reflects classical radical chain-breaking antioxidant activity by hydrogen atom transfer. Hence, the ORAC assay is limited to measuring hydrophilic chain-breaking antioxidant capacity against only peroxyl radicals. Interestingly, the ORAC assay is preferred to determine antioxidant capacity in foods, and is considered an indicator for potential biological activity, although the link between antioxidant capacity and protective health effects has yet to be completely established. Normally, the azo-compound employed in this assay generates peroxyl radicals. The most used azo-compounds include 2,2′azobis(2-amidinopropane) dihydrochloride (ABAP) and 2,2′-azobis(2,4-dimethylvaleronytril) (AMVN) [17]. According to this assay, the peroxyl radicals emitted by a generator react with a fluorescent sample, leading to a loss of fluorescence, which is measured with a fluorimeter. The addition of extra fluorescein is effective in addressing dynamic quenching by plasma [18]. 

Importantly, the ORAC assay uses the area under the curve technique in the presence and absence of the antioxidant. A standard reference antioxidant, typically Trolox, is used; thus, the ORAC values of the evaluated antioxidants are described in Trolox equivalents. A high-throughput assay has been developed to improve this method; it uses a multichannel liquid handling system coupled with a microplate fluorescence reader [19].

### 2.3. The FRAP Assay

The FRAP assay is a simple and fast method that is used to assess the antioxidant power of samples [20]; however, this method cannot detect antioxidants that act by radical quenching. This colourimetric assay is based on the ability of antioxidants to reduce the ferric tripyridyl triazine (Fe^3+^-TPTZ) complex to the ferrous form at a low pH. The end-product (Fe^2+^-TPTZ) has an intense blue colour with absorption at 593 nm, which can be monitored using a diode-array spectrophotometer to estimate the sample’s antioxidant capacity in kinetic mode for 60 min at 37 °C [21]. The FRAP reagent (20 mM FeCl_3_·6H_2_O solution, 10 mM TPTZ solution in 40 mM and 300 mM acetate buffer in a ratio 1:1:10, *v*/*v*/*v*) has some limitations due to its preparation, which is time consuming, and it is not stable for a long period of time [22]. A modification of the FRAP assay that employs Cu rather than Fe ions is called CUPRAC; it is based on the reduction of Cu^2+^ to Cu^+^ by the combined action of reducing agents in the sample. Neocuproine (2,9-dimethyl-1,10-phenanthroline) is used to form chromophores with Cu^+^ that absorb light at 450 nm [23,24,25].

### 2.4. The TRAP Assay

The TRAP assay is based on the capacity to inhibit the reaction between peroxyl radicals and a target molecule by antioxidants [26]. In this assay, oxygen molecule consumption in the peroxidation process triggers the thermal decomposition of ABAP. Thus, the TRAP assay could be considered a trapping method, and it has been widely applied to evaluate antioxidant capacity directly [27]. In this assay, the thermal decomposition of the water-soluble azo-compound (i.e., ABAP) generates peroxyl radicals at a known steady rate, which are monitored through a linear decrease in the R-phycoerythrin (R-PE) chemiluminescence quenching probe over time using a fluorimeter (excitation at 495 nm and emission at 575 nm). When the sample is added to the reaction mixture, the antioxidants protect R-PE from fluorescence decay. The length of the lag phase is used to estimate TAC directly [28]. This method is relatively more complex (i.e., requires a fluorimeter) and time-consuming than the above-mentioned assays. The TRAP assay can also use peroxyl radicals generated from 2,2′-azobis(2-amidinopropane) dihydrochloride (AAPH) and the peroxi-materials contained in plasma or other biological fluids [28]. After adding AAPH to the sample, the oxidative materials are monitored by measuring the oxygen consumed during the reaction. In this induction period, oxidation can be inhibited by the presence of antioxidants present in the plasma sample. The length of the induction period (lag phase) is compared with that of an internal standard (e.g., Trolox) and then quantitatively related to the plasma TAC. For example, by using the TRAP assay, Kharb [29] reported that serum TRAP experimental values were significantly higher in patients with pre-eclampsia compared with sex- and age-matched controls. Like the ORAC assay, the TRAP assay employs a fluorimeter that, despite its high sensitivity and high specificity, limits its use, potentially making it more tedious and requiring more sophisticated instruments. In fact, the TRAP assay is relatively complex and time-consuming to perform, and requires a high degree of expertise and know-how. In addition, this assay has been criticised because it employs a non-physiological source of OS (i.e., water-soluble peroxyl radicals).

Researchers have claimed that different exogenous factors regulate TAC values, including, among others, age, especially in the paediatric and aged populations [30,31,32], and exercise [33], with differential effects of acute and chronic status in trained athletes and sedentary subjects [33]. The hormonal influence on TAC has been less reported or has been underestimated, even though several endocrine and metabolic diseases are characterised by OS, including the defective function of the pituitary-dependent axes and in male infertility [34,35,36,37]. Several studies have been published on the role of OS in the development of metabolic syndrome, and especially its cardiovascular complications: much evidence supports this pathophysiological mechanism, which has also been linked to carcinogenesis [38,39,40]. In the scenario of a genetic pre-disposition to insulin resistance (IR), induced by excessive caloric intake or a sedentary lifestyle, researchers have hypothesised that there is diet-induced OS [41,42,43]. Therefore, TAC values need to be interpreted correctly and correlated with the specific clinical context to develop an appropriate treatment.

## 3. Perspective on Future Clinical Implication and Conclusions

OS has been widely investigated as a mechanism underlying the aetiology, clinical course and complications of several diseases. It can be evaluated by directly measuring oxidants in leukocytes and platelets by flow cytometry; the enzymatic components that determine the redox status; and more commonly, by the products of lipid, protein and nucleic acid oxidation [44]. The last category includes malondialdehyde, oxidised lipoproteins, nitrosamines, hexanoyl-lysine, nitro-tryptophane, 8-deoxyguanosine, thymidine glycol and others, as reviewed previously [45,46,47,48,49,50,51,52,53]. However, these indexes are not routinely used in clinical practice, nor are they included in a validated and standardised protocol. Nevertheless, antioxidants, both natural and nutraceutical/pharmacological, are administered extensively, often without a previous evaluation of the redox status of the specific condition at the cellular, tissue and whole body levels. Therefore, the search for simple and reproducible methods is still open, with the purpose of obtaining more complete knowledge of the pathophysiological picture of illnesses and determining biomarkers that help with the prognosis and therapeutic monitoring.

Here we discuss two of the most investigated clinical conditions characterised by increased OS: metabolic syndrome and infertility. Supplementation with antioxidants is employed extensively in patients with these conditions, and there is a need to search for appropriate biomarkers.

Interestingly, OS could play a role in metabolic syndrome-related manifestations that contribute to IR. Several dietary regimens with high levels of natural antioxidants have been proposed for supplementation [54,55]. We have already investigated the effects of dietary antioxidants on IR in subjects with obesity. Specifically, we compared a hypocaloric diet and a personalised hypocaloric diet enriched with natural antioxidants (with 800–1000 mg antioxidants per day from fruit and vegetables), alone or in combination with metformin. We found that, despite a similar body mass index (BMI) reduction, only the subjects that received the personalised antioxidant-enriched diet showed a decrease in HOMA (Homeostatic Model Assessment of Insulin Resistance) and the insulin peak after the glucose load was reached [56]. These findings suggest that a hypocaloric diet enriched with antioxidants is a better therapeutic approach.

Fresh fruit and vegetables are not the only natural antioxidants in the diet. In fact, previous studies have shown that a diet enriched with tree nuts is able to reduce glycated haemoglobin (HbA1c) in patients with diabetes [57]. This positive result is probably related to the antioxidant properties of such a diet. In preliminary studies, we demonstrated the superiority of a diet enriched with mixed dried fruits (20 g almonds and 30 g tree nuts) or with fruit and vegetables on IR [58]. Taken together, these preliminary data suggest that there is a differential metabolic response to various natural antioxidant-enriched diets, although the pathophysiological mechanisms remain to be elucidated.

Different bariatric surgical techniques—including biliopancreatic diversion, gastric bypass and mini-gastric bypass, recommended when class 3 obesity cannot benefit from diet and exercise alone—can induce dissimilar effects on antioxidant balance [59,60,61]. In a previous study, we evidenced a marked reduction in the lipid antioxidant coenzyme Q10 due to lipid malabsorption [13]. The mechanisms leading to this reduction (weight loss per se, lipid malabsorption and metabolic or hormonal variations) are not yet clear. We also suggest that these data, which could indicate a reduction in OS and therefore a reduction in compensatory antioxidant response, appear to be a promising index. However, from a long-term perspective, it could be a mechanism that contributes to the well-known phenomenon of weight regain. Therefore, assessment of TAC could be considered as a prognostic marker. Either way, we propose that TAC should be determined in patients who are undergoing bariatric surgery, even though this is not a routine measure recommended by the clinical guidelines [62]. When there is a persistent reduction in TAC, we recommend providing the patient with personalised antioxidant supplementation. Further investigations could provide insight into the differential patterns of specific antioxidants that can contribute to the antioxidant power in serum. 

Another clinical condition in which OS is considered to be a crucial part of the pathophysiological mechanism is infertility in men and women [63]. Oxidants do play physiological roles in fertility, but their excessive production can induce OS, which is an accepted mechanism for infertility [64]. Sperm cells are especially susceptible to OS damage due to the characteristics of their membrane and the lack of cytoplasm, which usually contains several enzymatic antioxidants (e.g., SODs). They are exposed to extensive oxidant-induced effects, including reduced membrane integrity (and therefore motility) and damage to proteins (and therefore structure and energy production) and DNA (with fragmentation and, ultimately, reduced fertility). 

Moreover, TAC is one of the most studied parameters of the antioxidant capability of seminal plasma. TAC is higher in fertile compared with infertile subjects; similarly, healthy sperm donors exhibited higher TAC versus patients with different seminal pictures (oligo- or asthenozoospermia, or both combined) [65,66]. These observations, together with the morphofunctional sperm alterations (mainly lipoperoxidation of membranes and DNA fragmentation) indicate very high OS. It has been reported that 30–80% of infertility cases are due to increased oxidant levels or low seminal plasma TAC [64]. More recently, researchers have proposed evaluating the balance between these two factors by evaluating the oxidation-reduction potential of semen [67]. Patients with normozoospermia have better parameters than those harbouring pathological conditions. We previously demonstrated that in patients with varicocele, a common state associated with male infertility, surgical treatment increased seminal plasma TAC [68]. The evaluation of TAC has been employed to establish the possible damage caused by spermatozoa refreezing techniques in assisted reproduction [69].

To contribute to this field, in this review, we want to shed light on the important role played by TAC. In fact, determining TAC is an inexpensive and simple method used to evaluate the antioxidant components, including non-protein and non-enzymatic small chain-breaking substances, in the plasma, but also in other biological fluids [70,71]. We recommend employing a TAC assay (i.e., TEAC/ABTS) to estimate the antioxidant levels in samples of human biological fluids. It should also be noted that a TAC assay does not provide information on the nature of the compounds, but it can be useful to evaluate synergistic interactions between antioxidants. Moreover, it has the advantage of providing a kinetic evaluation of rapid and slow components of the antioxidant reaction because there is a progressive enrolment of different antioxidants (small non-protein and non-enzymatic chain-breaking substances as the first line of defence and a subsequent protein response, including albumin and the above-mentioned enzymatic antioxidants) [72].

The results of a TAC assay should be carefully interpreted in the specific clinical context, and possibly, together with others parameters of oxidative damage (e.g., a SOD assay activity) and hormone quantification (e.g., dehydroepiandrosterone and testosterone), as hormones seem to influence antioxidants levels [73]. Thus, the combination of TAC evaluation with other assays of specific antioxidants and markers of oxidative damage would provide a comprehensive picture of the antioxidant status of a subject. In fact, a low TAC value can indicate a stable clinical condition, an inability to respond to the increasing production of radicals, or high antioxidant consumption due to OS.

In summary, we highlight four clinical implications of measuring TAC: (1) it can increase knowledge regarding the pathophysiology and clinical course of illnesses that involve OS; (2) it can serve a diagnostic and prognostic role based on the evaluation of the redox status; (3) it can inform the appropriate treatment when endogenous antioxidants are present at inadequate levels; and (4) it can be used to monitor the response to therapy. Based on these biochemical considerations and clinical studies, when appropriate, we recommend providing natural or chemical antioxidants as a complement to the ordinary pharmacological treatment of diseases that involve OS.

## Figures and Tables

**Table 1:** Several low-molecular weight “chain-breaking” plasma antioxidants and their respective sources, average concentration range and solubility. The antioxidants, such as vitamin C, E and A, are presently considered to be the main exogenous antioxidants ingested with diet. Glutathione (GSH), reduced by GSSG reductase (GR) at the expense of NADPH, and uric acid are considered the main endogenous antioxidants. For reference see [4] and references citied therein.

Antioxidant	Source	Range	Solubility
Vitamin C	Exogenous (diet)	0.4–1.5 mg/dL	Water-soluble
Vitamin E	Exogenous (diet)	7.4–23.5 mg/L	Fat-soluble
Vitamin A	Exogenous (diet)	0.22–0.62 mg/L	Fat-soluble
Uric acid	Purine catabolism	2.5–8 mg/dL	Water-soluble
GSH	GSSG reductases	60–80 μmol/L	Water-soluble

**Table 2:** The main features of the different techniques available to detect antioxidant activity. Methods include both those determining antioxidant enzymes (e.g., CAT) and oxidation products (e.g., LPO) in vivo and those determining antioxidants in vitro (e.g., TEAC).

Assay ^1^	Probe ^2^	Wavelength (λmax)	Method	Endpoint
TEAC	ABTS	Absorbance (734 nm)	Indirect	Lag-phase
ORAC	ABAP	Fluorescence (λex 495 nm)	Direct	Fixed time
FRAP	Fe-TPTZ	Absorbance (593 nm)	Indirect	Varies times
CUPRAC	Neocuproine	Absorbance (450 nm)	Indirect	Time
TRAP	R-PE	Fluorescence (λex 495 nm)	Direct	Lag-phase
DCHFT	DCHF-DA	Fluorescence (λex 502 nm)	Intracellular	Staining
NBT-T	NBT	Absorbance (540 nm)	Intracellular	Staining
DPPH-SA	DPPH	Absorbance (517 nm)	In vivo	% inhibition
SOD	Pyrogallol	Absorbance (420 nm)	In vivo	Fixed time
RP-m	K_3_Fe(CN)_6_/FeCl_3_	Absorbance (700 nm)	In vitro	Time
TBA-m	TBA	Absorbance (552 nm)	In vitro	Time
DMPD-m	DMPD	Absorbance (505 nm)	In vitro	Time
GSH	Ellman’s reagent	Absorbance (412 nm)	In vivo	standard curve
CAT	H_2_O_2_	Absorbance (240 nm)	In vivo	Time
LPO	MDA	Absorbance (532 nm)	In vivo	TBARS/mg protein

^1^ TEAC: trolox equivalent antioxidant capacity; ORAC: oxygen radical absorbance capacity; FRAP: ferric reducing ability of plasma; CUPRAC: cupric reducing antioxidant capacity; TRAP: total radical trapping antioxidant parameter; DCHF-T: dichlorodihydrofluorescein test; NBT-T: Nitroblue tetrazolium test; DPPH-SA: DPPH scavenging activity; SOD: superoxide dismutase activity; RP-m: Reducing power method; TBA-m: Thio-barbituric acid method; DMPD-m: DMPD method; GSH: Reduced glutathione estimation; CAT: Catalase activity; LPO: Lipid peroxidation assay. ^2^ ABTS: 2,2′-azino-bis(3-ethylbenzothiazoline-6-sulfonic acid); ABAP: 2,2′-azobis-amidinopropane; Fe-TPTZ: ferric tripyridyl triazine; Neocuproine: 2,9-dimethyl-1,10-phenanthroline; R-PE: R-Phycoerythrin fluorescent protein; DCHF-DA: 2,7-dichlorodihydrofluorescein diacetate; NBT: Nitroblue tetrazolium; DPPH: 1,1-diphenyl-2-picrylhydrazyl; Pyrogallol: benzene-1,2,3-triol; K_3_Fe(CN)_6_/FeCl_3_: potassium ferricyanide/ferric chloride; TBA: thiobarbituric acid; DMPD: N,N-dimethyl-p-phenylene diaminedihydrochloride; Ellman’s reagent: 5,5′-dithiobis-2-nitrobenzoic acid; H_2_O_2_: hydrogen peroxide; MDA: malondialdehyde; TBARS: thiobarbituric acid reactive substances.

## Data Availability

Not applicable.
